# The clinical features and prognostic outcomes of primary mediastinal yolk sac tumors: A retrospective analysis of 15 rare cases from multiple centers

**DOI:** 10.1097/MD.0000000000026480

**Published:** 2021-07-23

**Authors:** Li Qin, Menglin Zhu, Tiejun Wang, Hongli Wang, Qin Pan, Du He

**Affiliations:** a Department of Obstetrics and Gynecology, The Central Hospital of Enshi Autonomous Prefecture, Enshi, China; b Department of Oncology, The Central Hospital of Enshi Autonomous Prefecture, Enshi, China; c Department of Oncology, Hubei Cancer Hospital, Wuhan, China; d Department of Pathology, Minda Hospital of Hubei Minzu University, Enshi, China; e Department of Obstetrics and Gynecology, Jingzhou Central Hospital, The Second Clinical Medical College of Yangtze University, Jingzhou, China.

**Keywords:** clinical characteristics, primary mediastinal yolk sac tumor, prognosis, treatment

## Abstract

Primary mediastinal yolk sac tumors (PMYSTs) are a rare occurrence. As such, the clinicopathological features, treatment, and prognosis, of this disease still remain unclear. In this study, we aimed to provide further information relating to this rare malignancy in order to facilitate the creation of more specific clinical guidelines for the diagnosis and treatment of patients with PMYSTs.

In this retrospective study, we recruited 15 patients who had been diagnosed with PMYST from four medical institutions to create a population-based cohort. We then used Kaplan-Meier analysis and the log-rank test to investigate and compare overall survival (OS) and progression-free survival (PFS).

A total of 15 cases were identified. The mean age was 27.3 years (range: 19–34 years). The estimated 1- and 2-year PFS rates were 66.7% and 60.0%, respectively. The 1- and 2-year OS rates were both 73.3%. Computer tomography scans revealed tumors were located in the anterior middle mediastinum (5 cases), the anterior superior mediastinum (1 case), the left anterior mediastinum (3 cases), and the right anterior mediastinum (6 cases). Of the 15 patients receiving extended resections, the majority (40.0%) underwent tumor resection, partial pericardiotomy, pulmonary wedge resection, and mediastinal lymphadenectomy. R0 resections were achieved in eleven patients. Four patients underwent R2 resection and experienced postoperative complications, including pneumonia (2 cases), atelectasis (1 case), and bronchopleural fistula (1 case). Four patients developed postoperative lung metastasis. Three patients died due to progressive diseases. Disease recurred in all patients at a median of 8.0 months (range: 6.0–11.0 months).

PMYST is a rare but highly malignant tumor with a poor prognosis. Tumor resection, with optimal extended surgical management, may provide patients with the best chance of a cure although postoperative complications relating to the pulmonary systems should be treated with caution.

## 1 Introduction

Primary extragonadal malignant germ cell tumors (PEGCTs) are rare and are characterized by their localization along the midline of the body, including the mediastinum, retroperitoneum, coccyx, and central nervous system.^[1]^ Due to their location, PEGCTs present with distinctive clinical, biological, and prognostic characteristics. Malignant mediastinal germ cell tumors are very rare and account for only 1% to 4% of all PEGCTs.^[2]^ Of these mediastinal tumors, mature teratomas are the most common form of germ cell tumors, accounting for 70% to 75% of all mediastinal germ cell tumors; the second most common form are the primary mediastinal yolk sac tumors (PMYSTs).^[3]^ Previous studies have analyzed the clinical characteristics and prognosis of germ cell tumors located in the mediastinum, including seminoma, choriocarcinoma, teratoma, and embryonic carcinoma.^[4]^

PMYSTs are rare but highly malignant; patients with this form of tumor have a very poor prognosis. Although there are some reports relating to this condition in the existing literature, these publications relate to only small case series or individual case reports.^[5–7]^ Consequently, there is a significant lack of knowledge relating to the pathological diagnosis and surgical management of PMYST. There is no consensus with regards to a standard treatment for PMYST; for example, it is not clear whether or not it is necessary to perform retroperitoneal lymph node dissection, radiotherapy, or chemotherapy.^[6]^ Furthermore, biopsies taken from PMYSTs are particularly prone to misinterpretation. It is clear that the rarity of these tumors has restricted our ability to optimize therapeutic intervention, thus creating an urgent need to investigate the clinicopathological features and prognostic factors associated with PMYST.

In this retrospective study, we provide a detailed description of the clinical features of 15 Han Chinese subjects who were diagnosed with PMYST. We aimed to investigate the clinicopathological features, treatments, and prognostic factors, associated with these specific cases to guide clinical intervention and predict prognosis.

## 2 Material and methods

### 2.1 Case collection

Between January 2010 and December 2019, we recruited 15 cases of PMYST from several medical institutions (The Jingzhou Central Hospital, The Central Hospital of Enshi Autonomous Prefecture, The Minda Hospital, and The Hubei Cancer Hospital). The patient recruitment process was shown in Supplementary Figures 1. Of the 15 patients, 12 had been diagnosed with PMYST by histological analysis after surgery and three patients were diagnosed by needle core biopsy before surgery. All 15 patients underwent extended resection; post-surgical pathological analysis was consistent with biopsy results. The retrospective review of medical charts for the 15 patients was approved by the Institutional Research Ethics Committee of The Central Hospital of Enshi Autonomous Prefecture (No. 2020055), The Hubei Cancer Hospital (No. LLHBCH2020LW-001), The Minda Hospital (No. LLJCH2020LW-021), and The Jingzhou Central Hospital (NO.LLCHEAP2020LW-006). Because this study was retrospective, the Institutional Research Ethics Committee waived the need for patient consent in order for us to analyze medical charts. All patient information was strictly confidential, and our procedures were carried out in accordance with the Declaration of Helsinki.

### 2.2 Observational variables and survival outcome

We analyzed a number of clinical variables, including patient age, sex, symptoms, surgical management, and adjuvant treatment. Follow-up time was calculated from the date of admission for surgery, and the deadline for follow-up was August 30, 2020. Overall survival (OS) was defined as the time interval (in months) between the date of surgery and the date of death or censored. Progression-free survival (PFS) was defined as the time interval from initial surgery to tumor progression or death. Information relating to deaths, and the dates of any deaths, were obtained by telephone interviews or medical records.

### 2.3 Statistical analysis

According to different risk factors, we used the Kaplan-Meier method and the log-rank test to calculate OS and PFS curves. All analyses were conducted using the R statistical package (v.3.6.2; R Foundation for Statistical Computing, Vienna, Austria; https://www.r-project.org). A *P* value <0.05 was considered to be statistically significant.

## 3 Results

### 3.1 Clinicopathological features

**Figure 1:**
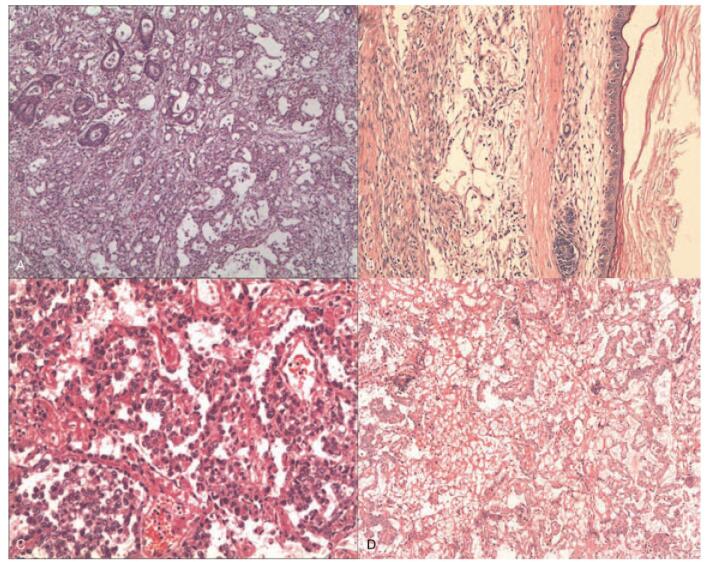
Pathology findings of patients diagnosed with PMYST. (A and C) Tumors are composed of clear, columnar epithelial cells arranged in sheets, cords and tubules structures. (A, H&E ×100; B, H&E ×200). (B) Immunostaining of alpha-fetoprotein (H&E ×100). (D) Histological features of Schiller Duval bodies. (H&E ×100).

Fifteen consecutive patients were treated for PMYST between January 2010 and December 2019 and recruited into this study. Fourteen of the patients were young men; case 12, however, was a young woman who did not have an ovarian yolk sac tumor before admission. Mean age was 27.3 years (range: 19–34 years). Fourteen patients presented with chest tightness or chest pain; 2 presented with dyspnea; 1 presented with asthma; and 1 patient presented with cough and chest pain and showed atypical intermittent fever. All patients exhibited markedly high levels of AFP before surgery; none had any significant medical problems in the past; mean AFP was 12105.2 ng/mL (range: 212.3–30825.0 ng/mL). Levels of human chorionic gonadotropin were within the normal range. The Ki67 proliferation index ranged from 3% to 70%. Immunohistochemical analysis revealed the positive expression of several biomarkers, including AFP (15/15), CK7 (10/10), CKL (13/13), and PLAP (6/6), and the negative expression of CK20 (2/2), CEA (14/14), HCG (6/6), and CD30 (13/13). EMA was positive in two (2/5) patients. Hematoxylin and eosin staining results, taken from pathological sections, are shown in Figure 1. Each risk subgroup represented a distinct prognosis and the OS in the 2 subgroups was accurately separated by Ki67 (Supplementary Figures 2). Computer tomography (CT) scans revealed the location of tumors in the anterior middle mediastinum (5 cases), anterior superior mediastinum (1 case), left anterior mediastinum (3 cases), and right anterior mediastinum (6 cases). The mean tumor size was 7.56 cm (range: 3.6–11.0 cm). Patient characteristics are summarized in Table 1 and Table 2.

**Table 1:** Clinical features, treatments, and prognostic outcomes for 15 patients diagnosed with PMYST.

Patient ID	Sex/age, y	BMI, kg/m^s^	Clinical symptoms	History	Site	Size, cm	Surgery	Adjuvant therapy	Metastasis	Follow-up, mo
Case 1	Male/30	26.32	Chest pain	1 mo	LAM	3.6 × 2.2 × 1.9	TPPL	EP	None	Alive, 57
Case 2^∗^	Male/33	25.78	Chest tightness	2 wk	ASM	10 × 7.5 × 6.7	TPNL	BEP	Pulmonary	Dead, 8
Case 3	Male/29	23.24	Dyspnea/chest pain	2 wk	LAM	8.5 × 7.6 × 7.6	TPNL	EP	None	Alive, 31
Case 4	Male/24	27.81	Chest pain	6 mo	LAM	5.6 × 6.3 × 6.5	TPPL	BEP	None	Alive, 25
Case 5^∗^	Male/21	22.37	Chest tightness	3 mo	RAM	2.6 × 3.8 × 1.2	TPPPL	BEP	None	Alive, 46
Case 6	Male/19	19.76	Chest pain/asthma	2 wk	AMM	6.7 × 8.2 × 7.5	TPL	BEP	None	Alive, 61
Case 7	Male/28	27.67	Chest pain	6 wk	RAM	4.7 × 6.8 × 7.1	TPL	BEP	None	Dead, 12
Case 8^∗^	Male/22	23.31	Dyspnea/chest pain	1 mo	RAM	6.5 × 6.3 × 7.5	TETL	BEP	Pulmonary	Dead, 7
Case 9	Male/29	24.67	Shortness of breath	10 days	AMM	7.5 × 6.3 × 5.5	TPPL	EP	None	Dead, 15
Case 10	Male/31	23.82	chest pain	2 wk	AMM	11.0 × 4.7 × 7.5	TPL	BEP	None	Alive, 36
Case 11^∗^	Male/32	24.56	Chest tightness	2 mo	RAM	5.8 × 5.6 × 8.0	TETL	BEP	None	Alive, 22
Case 12	Female/30	19.72	Cough/chest pain	2 wk	AMM	6.8 × 6.5 × 7.2	TPL	BEP	Pulmonary	Dead, 11
Case 13^∗^	Male/34	23.61	Intermittent chest pain	6 mo	RAM	5.6 × 6.1 × 5.5	TPPL	EP	None	Alive, 28
Case 14	Male/25	22.18	Chest tightness/chest pain	3 mo	AMM	7.6 × 5.6 × 6.2	TPPL	BEP	Pulmonary	Dead, 6
Case 15^∗^	Male/22	26.64	Chest tightness	6 wk	RAM	10.8 × 7.6 × 6.4	TPPL	BEP	None	Alive, 44

Notes. Cases that underwent needle biopsy. AMM = anterior middle mediastinum, ASM = anterior superior mediastinum, BEP = cisplatin, etoposide, and bleomycin, BMI = body mass index, EP = etoposide and cisplatin, LAM = left anterior mediastinum, RAM = right anterior mediastinum, TETL = tumor resection, extended thymectomy, and mediastinal lymphadenectomy, TPL = tumor resection, partial pericardiotomy, and mediastinal lymphadenectomy, TPNL = tumor resection, partial pericardiotomy, phrenic nerve resection, and mediastinal lymphadenectomy, TPPL = tumor resection, partial pericardiotomy, pulmonary wedge resection, mediastinal lymphadenectomy, TPPPL = tumor resection, partial pericardiotomy, pulmonary wedge resection, partial superior vena cava resection, and mediastinal lymphadenectomy.

**Table 2:** The expression of immunohistochemical markers in 15 patients diagnosed with PMYST.

Patient ID		Immunohistochemical markers												
	CK7	EMA	CK20	CEA	TTF-1	AFP	CKL	CD30	PLAP	HCG	GPC-3	CKH	CD117	Ki67
Case 1	+	+	—	—	—	+	/	/	/	/	/	/	/	—
Case 2	/	/	/	—	/	+	+	—	+	/	/	/	/	+
Case 3	+	/	/	/	/	+	+	—	/	/	/	+	/	+
Case 4	/	/	/	—	/	+	+	—	+	/	/	/	/	—
Case 5	/	+	—	—	—	+	+	—	+	—	/	/	/	/
Case 6	/	/	/	—	/	+	/	/	/	/	/	/	/	+
Case 7	+	/	/	—	/	+	+	—	+	/	/	/	/	+
Case 8	+	/	/	—	/	+	+	—	/	—	/	/	/	/
Case 9	/	/	/	—	/	+	+	—	+	—	/	/	—	+
Case 10	+	/	/	—	/	+	+	—	/	—	/	/	/	/
Case 11	+	—	/	—	/	+	+	—	+	/	/	/	/	+
Case 12	+	—	/	—	/	+	+	—	/	—	/	/	/	—
Case 13	+	/	/	—	/	+	+	—	/	/	/	+	/	+
Case 14	+	/	/	—	/	+	+	—	/	—	+	/	/	+
Case 15	+	—	/	—	/	+	+	—	/	/	/	+	/	+

Notes: + = Positive, − = negative, / = missing data.

### 3.2 Surgical treatment and adjuvant therapy

**Figure 2:**
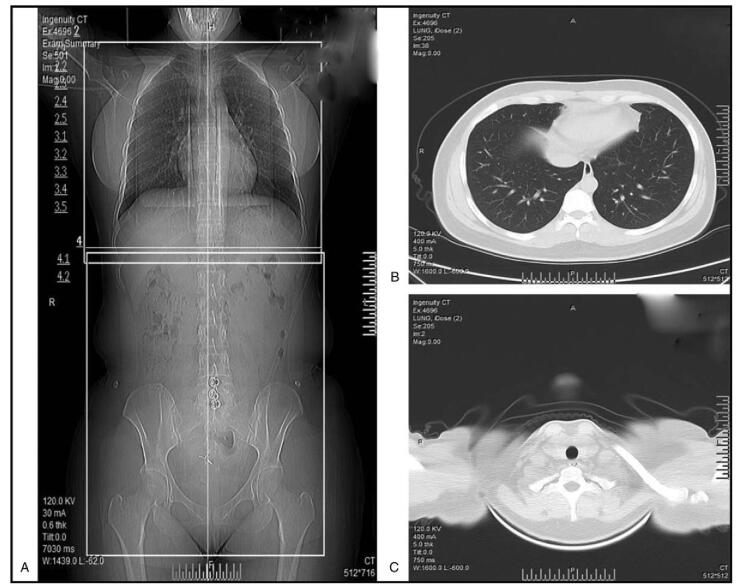
Computed tomography (CT) imaging of patients diagnosed with PMYST before surgery and postoperative chemotherapy (Case 5). (A) Computed tomography scan on admission. (B) Computed tomography scan of the thorax. (C) Computed tomography scan of the pelvic cavity.

**Figure 3:**
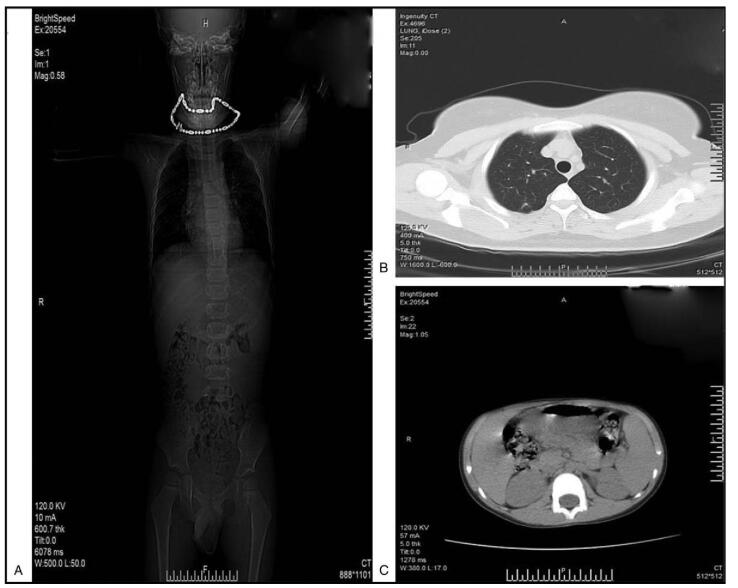
Computed tomography (CT) imaging of patients diagnosed with PMYST after surgery and postoperative chemotherapy (Case 1). (A) Computed tomography scan on admission. (B) Computed tomography scan of the thorax. C. Computed tomography scan of the pelvic cavity.

Following evaluation, all patients underwent extended resection. Six cases underwent tumor resection, partial pericardiotomy, pulmonary wedge resection, and mediastinal lymphadenectomy (TPPL); 15 cases underwent tumor resection, partial pericardiotomy, and mediastinal lymphadenectomy; 4 cases underwent tumor resection, extended thymectomy, and mediastinal lymphadenectomy (TPL); 2 cases underwent tumor resection, extended thymectomy, and mediastinal lymphadenectomy (TETL); and 2 cases underwent tumor resection, partial pericardiotomy, phrenic nerve resection, and mediastinal lymphadenectomy (TPNL). One case underwent tumor resection, partial pericardiotomy, pulmonary wedge resection, partial superior vena cava resection, and mediastinal lymphadenectomy. All of these patients received platinum-based chemotherapy. The majority of patients received a BEP regimen, and 4 cases received an EP regimen. Postoperative examination showed that there had been no lymph node metastasis in any of the patients receiving mediastinal lymphadenectomy. We ensured that we removed as much of each tumor as possible. R0 resections were achieved in eleven patients. Four patients underwent R2 resection and experienced postoperative complications, including pneumonia (2 cases), atelectasis (1 case), and bronchopleural fistula (1 case). Typical preoperative and postoperative computer tomography results are shown in Figure 2 and Figure 3.

### 3.3 Follow-up and survival outcome

**Figure 4:**
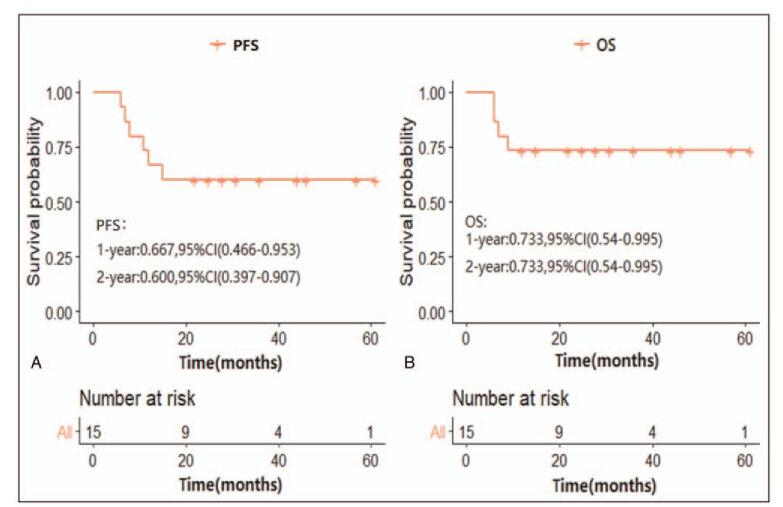
Kaplan-Meier estimates for patients with PMYST after treatment. (A) Kaplan-Meier survival curves for overall survival. (B) Kaplan-Meier survival curves for progression-free survival.

The estimated 1- and 2-year PFS rates were 66.7% and 60.0%, respectively, whereas the 1- and 2-year OS rates were 73.3% (Fig. 4). Four patients developed postoperative lung metastasis. All cases of disease recurrence occurred in a median of 8.0 months (range: 6.0–11.0 months). Complete remission was achieved in one patient; however, this patient died after 2 months due to pulmonary infection. The remaining 3 patients died due to progressive diseases.

## 4 Discussion

PMYST is a rare disease that typically arises in midline locations in adults, particularly the anterior mediastinum, the retroperitoneum, and the pineal and suprasellar regions.^[6]^ Based on histological criteria, PEGCTs are classified as either seminomatous primary mediastinal yolk sac tumor (S-PMYST), non-seminomatous primary mediastinal yolk sac tumor (NS-PMYST), mature teratomas, or immature teratomas. According to this classification method, PMYSTs are a form of non-seminomatous PEGCT. PMYST is the second most frequent form of PEGCTs; these tumors occur predominantly in males and are relatively rare in females. Only a very limited number of studies have reported the clinicopathological and prognostic studies of PMYST. Thus, our understanding of this form of tumor is very limited and needs to be improved. To the best of our knowledge, this study features the largest number of PMYST cases thus far. Our study focused on a Han Chinese cohort of patients with PMYST that was recruited from several centers. Our aim was to advance knowledge relating to this rare form of tumor and thus help to guide clinical diagnosis and management.

Our own records, taken over the last decade, indicate that the incidence of PEGCTs is approximately 3% of all germ cell tumors; this figure is consistent with previous studies.^[6,8–10]^ Presently, the most common form of diagnosis is the application of needle core biopsy, computed tomography, and serological indicators. However, due to the specific location of PMYST, and the atypical symptoms of the patient involved, biopsy is not necessarily an efficient or recommended procedure; imaging diagnosis appears to be more reliable.

According to the International Germ Cell Cancer Collaborative Group (IGCCCG), PMYST is associated with a poor prognosis with a 40% to 50% OS.^[11–13]^ The current form of treatment for patients with PMYST is chemotherapy; this can be combined with residual mass resection, carried out pre- or post-chemotherapy. In this study, patients who underwent needle core biopsy subsequently received pre-operative platinum-based chemotherapy and then underwent extended resection; all of the patients received postoperative platinum-based chemotherapy. We concluded that the estimated 1- and 2-year PFS rates were 66.7% and 60.0%, respectively; these figures are consistent with previous publications.^[6,8,9,14]^ There are several factors underlying the poor prognosis of these patients. For example, the bulky presentation of these types of tumors make them almost impossible to completely resect in patients in advanced stages.^[2,15]^ Furthermore, rapid growth and early metastasis should also be evaluated in an adequate manner, including lung, brain, liver, and bone, metastases.^[15]^ At present, there are no standards for the surgical management of these tumors; extended resection, however, does appear to be a routine procedure.

All of the patients in this study underwent extended resection, including partial pericardiotomy, pulmonary wedge resection, mediastinal lymphadenectomy, phrenic nerve resection, and mediastinal lymphadenectomy. Although lymph node dissections failed to identify metastasis, we suggest that routine lymph node biopsy is necessary, especially for patients undergoing extensive resection; this procedure can be used as an important basis for screening distant metastasis. Furthermore, disease recurrence (pulmonary metastasis) occurred in 4 patients between 6 and 11 months. In the present study, patients who underwent surgery, but experienced recurrence with pulmonary metastasis, did not live for >2 years. We speculate that patients who develop lung metastasis following chemotherapy are still at risk of distant metastasis. Moreover, lung function following lobectomy is poor; therefore, patients are prone to pulmonary infection, atelectasis, and other complications.

Due to our limited sample size, it was difficult to evaluate prognostic factors for our patients. We attempted to stratify the factors that may be closely related to the prognosis of tumor patients, including tumor size and the Ki67 proliferative index. We found that the Ki67 proliferative index may be a potential predictor for OS, consistent with previous studies.^[16–18]^ We hypothesized that a lower proliferative rate might contribute to a better prognostic outcome, thus providing practical and clinical information relating to prognosis for clinicians; this might allow targeted forms of therapy. A better understanding of molecular structure and different protein domains along with its regulation will provide evidence for precise target thereby controlling the proliferation rate correlated with decrease in the Ki-67 protein levels.^[19]^ However, this hypothesis needs to be confirmed in a larger population.

Nevertheless, the present study has several limitations that need to be considered. First, our study elucidated the epidemiology of YST in a single-center cohort of the Chinese Han population, further studies are needed to elucidate by the large population-based study. Second, we recruited 15 cases from several different centers over the space of 10 years. It is possible that our results may have been influenced by changes in clinical practice over this long time period. Third, our study focused on young men. It is now important to investigate the clinicopathological features and treatment options that relate to adult women and adolescents. Finally, this was a retrospective study that could not completely avoid data selection and measurement biases, it is necessary to validate the results from more prospective studies or multicenter studies in the future.

## 5 Conclusions

PMYST is a rare and highly malignant for of tumor that is associated with a very poor prognosis. However, cisplatin-based chemotherapy, followed by surgical resection of residual masses, may help to improve long-term survival. Predictive prognostic factors, and improved therapeutic options, are urgently needed for these Chinese Han patients. As more information accumulates for this rare form of tumor, it will be possible to create more standardized strategies for the management of this tumor.

## Acknowledgments

The authors thank all patients involved in this study.

## Author contributions

**Formal analysis:** Menglin Zhu, Qin Pan.

**Investigation:** Li Qin, Hongli Wang, Qin Pan.

**Methodology:** Li Qin.

**Resources:** Hongli Wang.

**Software:** Tiejun Wang.

**Supervision:** Tiejun Wang.

**Visualization:** Tiejun Wang.

**Writing – review & editing:** Du He.

## Supplementary Material

Supplemental Digital Content
