# Supplementary material for: The clinical features and prognostic outcomes of primary mediastinal yolk sac tumors: A retrospective analysis of 15 rare cases from multiple centers
Source: Medicine (Baltimore). 2021 Jul 23;100(29):e26480. doi: 10.1097/MD.0000000000026480 (PMC8294869; doi:10.1097/MD.0000000000026480)

**Supplementary Figures1. Flowchart for the inclusion of patients with PMYST**


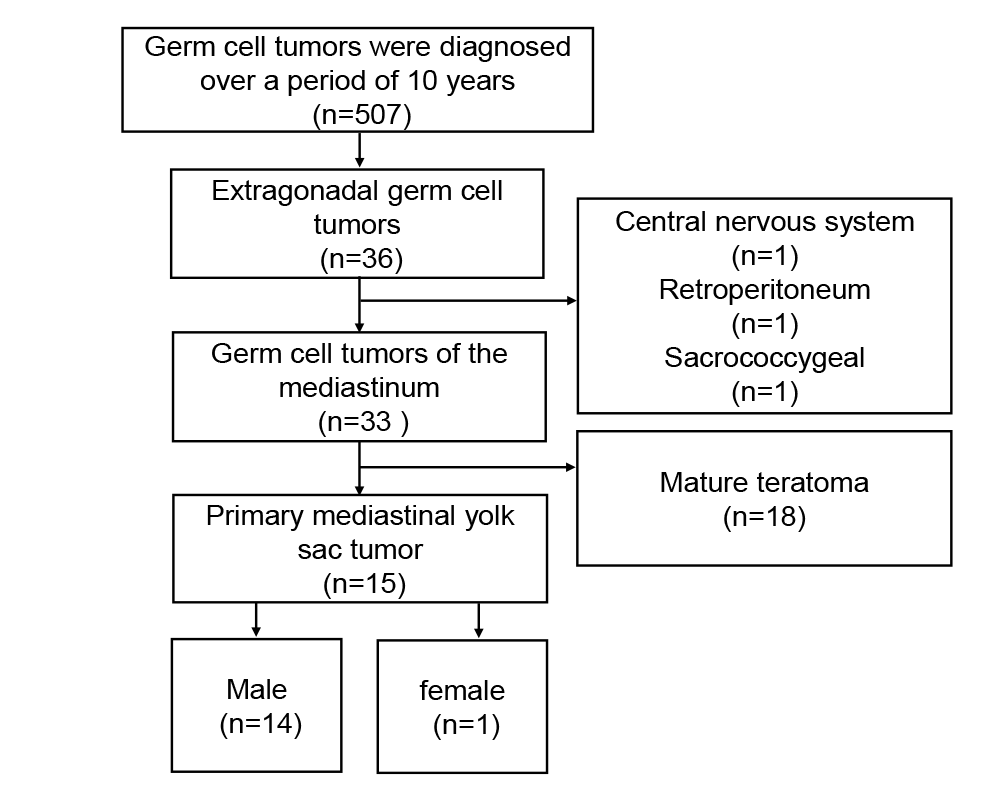


**Supplementary Figures2. Kaplan-Meier analysis of survival outcome for patients stratified by the Ki67 index**

**A. Kaplan-Meier analysis of overall survival stratified by Ki67 index**

**B. Kaplan-Meier analysis of progression-free survival stratified by Ki67 index**


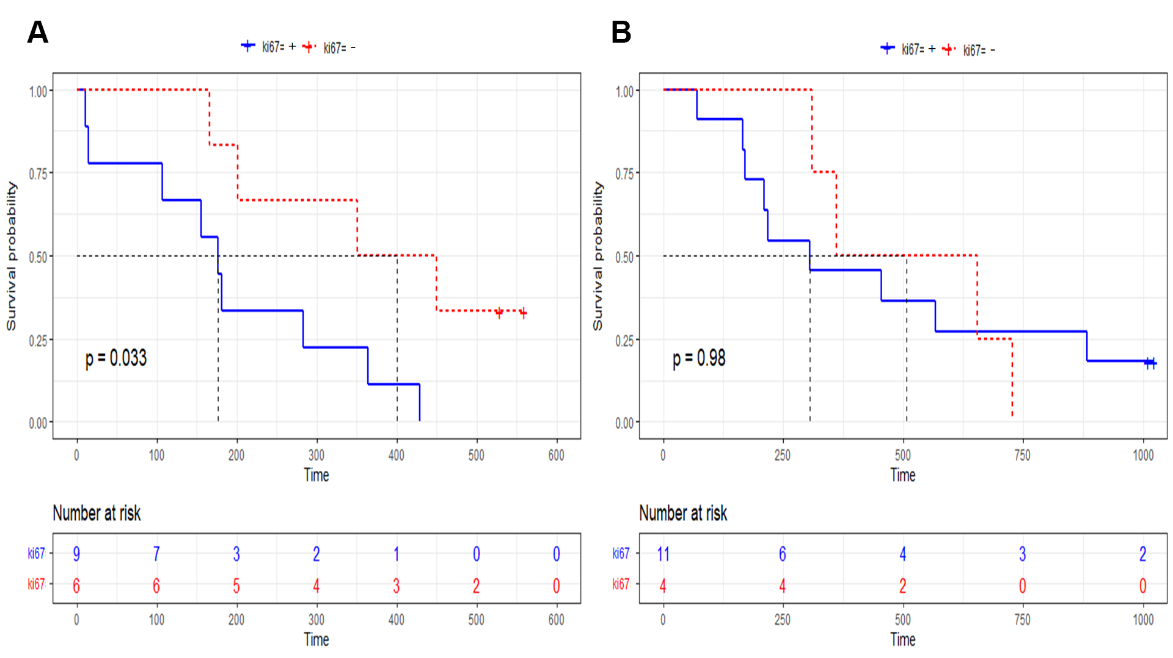

Supplement: Supplemental Digital Content [file medi-100-e26480-s001.doc]
